# Delayed Infection of Occipitocervical Fixation in a Patient with Achondroplasia: A Case Report and Review of Literature

**DOI:** 10.31661/gmj.v9i0.1906

**Published:** 2020-06-15

**Authors:** Milad Shafizadeh, Ehsan Fattahi, Sabra Rostamkhani, Mohsen Rostami, Alireza Khoshnevisan

**Affiliations:** ^1^Department of Neurosurgery, Shariati Hospital, Tehran University of Medical Sciences, Tehran, Iran; ^2^Department of Neurosurgery, Golestan University of Medical Sciences, Gorgan, Iran; ^3^Sports Medicine Research Center, Neuroscience Institute, Tehran University of Medical Sciences, Tehran, Iran

**Keywords:** Achondroplasia, Surgical Infection, Craniocervical

## Abstract

**Background:**

Infections are a major concern in fixation surgeries. Most of the infections could occur in the first three months after the operation.

**Case Report:**

We present a 45-year-old man who known case of achondroplasia who underwent craniospinal fixation and was presented to our clinic with surgical site infection after six years. His instruments were removed, and a halo vest was fixed for the patients. Accordingly, he received intravenous antibiotics, and during nine months’ follow-up, no any significant problems were found.

**Conclusion:**

Infection of instruments in spinal surgeries might be presented years after the surgery. Hence, it needs to be considered by surgeons in patients’ follow-ups.

## Introduction

Achondroplasia is an autosomal dominant disease with distinctive clinical symptoms (such as short-limbed dwarfism, craniofacial deformities, rhisomelia, and trident hand) resulting fromaa defect in enchondral bone formation. These patients usually have a large head with narrow foramen magnum and small craniocervical junction [1]. Patients with achondroplasia are susceptible to cervicomedullary compression, spinal canal stenosis (cervical and lumbar), syringomyelia, and spinal instability [2]. The craniocervical instability in these patients can be de novo or after C1 laminectomy (due to foramen magnum stenosis)[3]. The craniocervical junction has been the topic of interest for many clinicians over the past decades due to special features such as complex ligamentous and bony anatomy and representing the most mobile component of the spine axis [4] while harboring vital neural structures [5]. Occipitocervical fusion (OCF) has been widely used in the approach to patients with craniocervical instability with an acceptable rate of biomechanical stabilization and bone fusion [6]. To achieve early stabilization, reduce the need for post-operative immobilization and improve the fusion rate, instrumentation (use of screw and plates) for OCF has been suggested [6,7]. However, there are many reports on possible complications of this procedure, such as vascular insult, cerebrospinal fluid (CSF) leakage, and wound infection [6]. In this report, a patient with achondroplasia with progressive neurological symptoms is described who underwent OCF and presented to our clinic with instrumentation failure and infection four years after the surgery. We also discuss the surgical management of cervical instability in patients with achondroplasia and review the relevant literature.

## Case Presentation

**Figure 1:**
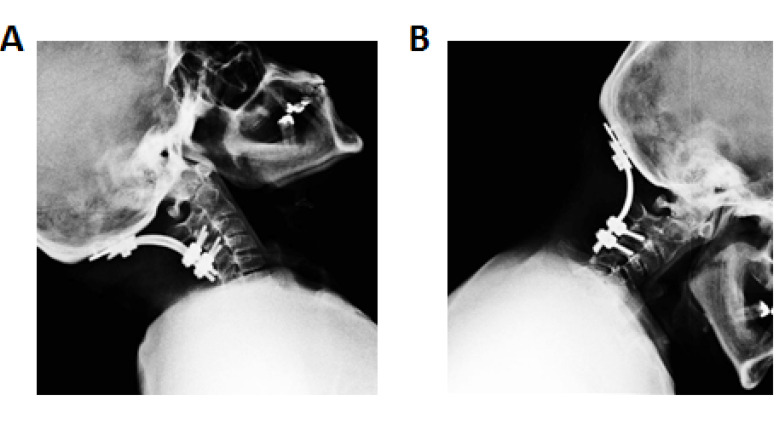
The flexion (**A**) and extension (**B**) views of the patient show halo sign around the lateral mass screws and instability at the craniocervical region.

 A 45-year-old man known case of achondroplasia was presented with a history of progressive headache and weakness of upper and lower extremities over the one year. He also had complained of paresthesia and gait disturbance since one month before admission. Neurological exam revealed the force of 4/5 at upper extremities and spastic paraparesis (proximal forces 3/5 and distal forces 4/5) and hypesthesia to pinprick and light touch. Deep tendon reflexes were increased, and plantar responses were extensor bilaterally. There were no bowel or bladder symptoms. Dynamic lateral x-rays and magnetic resonance imaging (MRI) were done, and occipitocervical instability was diagnosed. The patient underwent OCF with an occipital plate and lateral mass screws at C3 and C4. Gradually, over one year, all the symptoms were revealed, and the patient backed to his work. After six years, the patient was referred to our center with the complaint of wound discharge. Lab data including erythrocyte sedimentation rate, C-reactive protein, and complete blood count were investigated, blood and wound culture were provided, and further sepsis workups were done. According to our lab findings, no infection was found. Moreover, all the inflammatory findings were within the normal range. The patient was also complaining of recurrence of previous symptoms such as gain difficulty and hypesthesia over the last six months. A lateral dynamic x-ray was taken, which showed possible instability. Computed tomograph scan was done that showed the failure of the fusion construct (Figure-1). The patient underwent surgical exploration, and we found that all instruments are quite loose. All of them were removed, and a halo vest was fixed for the patients. The culture from instruments was negative, but the patient underwent eight weeks of intravenous (IV) antibiotic therapy (meropenem and vancomycin) following by eight weeks of oral therapy with clindamycin (600mg q8hours). The symptoms were improved, and the patient discharged. At three and nine months’ follow-ups, no significant problem were found.

## Discussion

Achondroplasia has been associated with a high incidence of cervical instability. Although many patients are initially asymptomatic, the instability of the upper cervical spine may progress to subluxation and dislocation; and subsequently lead to cervical myelopathy, quadriparesis, and possible death. OCF is a common procedure performed for occipitocervical instability [8]. Although this procedure has a high rate of success in most studies, it can also bring about various rare complications such as infection. Previous studies revealed that the rate of surgical site infection has been reported from 10% [9] to more than 35% [10] in different series. The infection is mostly managed by IV antibiotics and immobility. Mazur *et al*. conducted a study in which one patient developed an infected wound after OCF, which required instrumentation removal and long-term antibiotic therapy [11]. Also, surgical site infection up to one month after surgery has been reported in different studies that investigated the craniocervical fusion techniques, but to our knowledge, this is the first study, which presents a case of wound discharge, six years after surgery [12,13]. Hwang *et al*. reported the infection occurs in a patient 12 days after craniocervical fixation [14]. Patkar *et al*. also reported the occurrence of wound infection and implant failure in a patient six weeks after surgery that required instrumentation removal [12]. A common etiology for infection in the craniocervical region might be CSF leakage and meningitis, which superimposes on that. Adeolu *et al*. reported a patient with post-operative deep infection that was treated with parenteral antibiotics and wound dressing [15]. However, according to surgical notes, no dura injury happened during the first operation. Higher duration of the operation and posterior cervical approaches might be other predisposing factors of infection that were present in our case.

## Conclusion

 This report might further imply the importance of long-term follow-up of the patient who undergoes craniocervical fusion and shows that they are at risk of delayed infections.

## Conflict of Interest

 All the authors declare there were no any conflicts of interest.
